# Latent reservoirs in memory T cell subsets are linked to poor immune recovery in people living with HIV

**DOI:** 10.3389/fimmu.2025.1684924

**Published:** 2025-12-02

**Authors:** Selwyn Selva Kumar, John Paul Demosthenes, Madheswaran A., Milton P., Inbanathan A., Nagaraj V., Abi Manesh, Saravanabhavan Thangavel, Luke Elizabeth Hanna, Rajesh Kannangai, George M. Varghese

**Affiliations:** 1 Department of Infectious Diseases, Christian Medical College, Vellore, India; 2 Department of Clinical Virology, Christian Medical College, Vellore, India; 3 Department of Virology and Biotechnology, Indian Council of Medical Research (ICMR)-National Institute of Research in Tuberculosis, Chennai, India; 4 Center for Stem Cell Research (CSCR), a Unit of BRIC-Institute for Stem Cell Science and Regenerative Medicine (BRIC-in Stem, Bangalore), Christian Medical College, Vellore, Tamil Nadu, India

**Keywords:** viral latency, HIV, immune reconstitution, T-lymphocyte subsets, CD4-positive T-lymphocytes, highly active antiretroviral therapy

## Abstract

Highly active antiretroviral therapy (HAART) suppresses viral loads in 71% of people living with HIV globally, while failing to bring adequate immune reconstitution in nearly one-third of them. We hypothesize that the persistence of latent HIV reservoirs in specific memory T cell subsets contributes to impaired immune recovery. We conducted a case-control study to estimate differences in the HIV-1 proviral DNA across memory CD4-positive T cell subsets between participants with CD4 counts of over 500 cells/µL (immune responders or IRs) and those with counts of less than 350 cells/µL (immune non-responders or INRs) with sustained viral suppression. Latent HIV reservoirs (LRs) were detected in at least one memory T cell subset in 48.33% of total participants. Latent reservoirs were more frequent among INRs than among IRs (65.38% versus 35.29%, P = 0.02), particularly in the effector memory T cell subset (34.6% in INRs versus 8.8% in IRs, P = 0.02). Thus, despite long-term viral suppression with HAART, the persistence of latent reservoirs in memory T cells is associated with poor CD4-positive T cell recovery. Emerging classes of antiretroviral agents that target latent viral pools may enhance immune restoration and bring us closer to finding an HIV cure.

## 1 Introduction

Though highly active antiretroviral therapy (HAART) has had remarkable success in achieving viral suppression in approximately 71% of people living with HIV (PLWH) globally, it fails to achieve adequate immune reconstitution in a significant proportion (nearly one-third) (1, 2). Individuals on HAART with impaired immune recovery, termed immune non-responders (INRs), maintain low CD4-positive T cell counts despite the sustained suppression of viral load. The resultant subclinical immunodeficiency heightens the INRs’ risk of HIV-related and HIV-unrelated complications, accelerates the progression of acquired immunodeficiency syndrome (AIDS), and increases mortality (3, 4).

The underlying mechanism for poor recovery of CD4-positive T cells in INRs remains incompletely understood. Proposed explanations include persistent immune activation, immune exhaustion, virological factors, host genetic variability, and suboptimal thymic output (2). However, none of these factors fully explains the INR phenotype.

A defining feature of HIV pathogenesis is the establishment of latent reservoirs within resting memory CD4-positive T-cells, where the virus persists in a transcriptionally silent state. These reservoirs evade clearance by HAART and remain the principal barrier to curing HIV. Notably, latent HIV has been detected even after allogeneic hematopoietic stem cell transplantation from CCR5 wild-type donors, underscoring the resilience of these viral sanctuaries (5).

We hypothesize that the persistence of latent HIV reservoirs in specific memory CD4 -positive T cell subsets may be associated with impaired immune recovery in immune non-responders. Elucidating this link could provide critical insights into the pathogenesis of immune non-responsiveness and inform the development of targeted therapies aimed at both immune reconstitution and reservoir eradication.

## 2 Materials and methods

We conducted a case-control study to estimate differences in the HIV-1 proviral DNA across memory CD4-positive T cell subsets between IRs and INRs. INRs were defined as people living with HIV-1 who had been on HAART for at least two years, demonstrated sustained viral suppression, and had an absolute CD4-positive T-cell count of less than 350 cells/µL for at least two years prior to recruitment. IRs were defined as individuals with CD4-positive T cell counts consistently greater than 500 cells/µL for at least two years prior to recruitment. All participants who met the inclusion criteria and visited the clinic during the study period were randomly enrolled in the study. Exclusion criteria included individuals who were below 18 years of age, had HIV-2 infection, had a documented episode of detectable viremia during the past two years, or had difficult venipuncture.

Ethical approval was obtained from the Institutional Review Board of Christian Medical College, Vellore (IRB Min. No. 13143). Sixty participants were recruited between March 2021 and August 2021 from the National AIDS Control Organization (NACO) ART Centre and the Infectious Diseases outpatient department of Christian Medical College and Hospital, Vellore. After obtaining informed written consent, 10 mL of peripheral blood was collected from each participant.

We collected data on epidemiological factors, co-morbidities, duration of HIV-1 infection, duration and type of HAART regimen, history of opportunistic infections, and metabolic complications. Peripheral blood mononuclear cells (PBMCs) were isolated from blood samples, and CD4-positive T cells were sorted based on established surface markers into central memory (TCM), transitional memory (TTM), and effector memory (TEM) subsets using flow cytometry.

### 2.1 PBMC separation

Peripheral blood mononuclear cells were isolated from leukapheresis samples using Ficoll density gradient centrifugation and stored at −80 °C for 2.5 years. For analysis, the samples were thawed at 37 °C in a water bath, washed, and assessed for viability using the trypan blue dye exclusion method. Only samples with a viability greater than 60% were included for further analysis.

### 2.2 Cell surface staining

To identify and sort CD4^+^ T-cell subsets, cells were stained with the fluorochrome-conjugated monoclonal antibodies listed in **Table 1** (all from BD Biosciences).

**TABLE 1:** Panel of fluorochrome-conjugated monoclonal antibodies used for CD4^+^ T-cell subset identification and sorting.

Marker	Fluorochrome	Clones
Fixable Viability Stain	APC-H7	–
CD3	Brilliant Violet 510 (BV510)	UCHT1
CD4	APC-R700	SK3
CD45RO	APC	UCHL1
CCR7	PE-Cy7	2-L 1-A
CD27	Brilliant Violet 421 (BV421)	M-T271

For staining, 4 × 10^6^ to 10 x 10^6^ cells were incubated with the antibody cocktail in staining buffer (PBS + 2% FBS) for 30 min at 4 °C in the dark. After staining, cells were washed twice with staining buffer and resuspended in PBS containing 2% FBS for flow cytometric acquisition and sorting.

Prior to acquisition, cells were incubated with Fixable Viability Stain (APC-H7) according to the manufacturer’s protocol to exclude non-viable cells. Live (APC-H7^−^) cells were gated for further analysis and sorting.

### 2.3 Flow cytometric sorting using FACS Aria III

**Figure 1:**
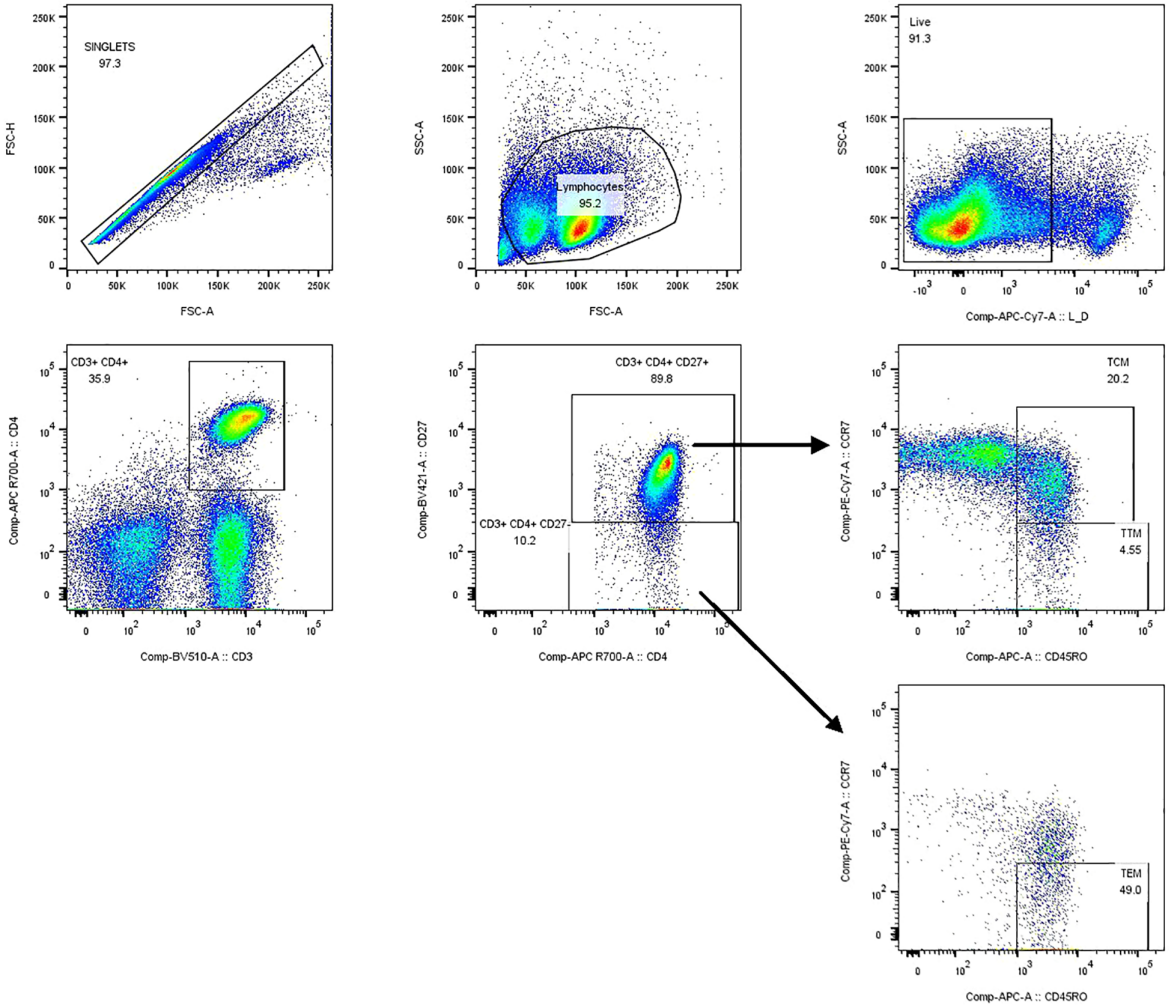
Memory CD4 T-cell subsets sorting strategy.

Cell sorting was performed on a BD FACS Aria III instrument. Compensation controls were prepared using single-stained beads or cells to adjust for spectral overlap. The gating strategy was as follows: Singlet cells were selected using FSC-A vs. FSC-H. Live cells were identified as APC-H7-negative. T cells were gated as CD3^+^. CD4^+^ helper T cells were gated within the CD3^+^ population. Memory subsets were identified using CD45RO, CCR7, and CD27 expression: Central memory (CD45RO^+^ CCR7^+^ CD27^+^), Transitional memory (CD45RO^+^ CCR7^−^ CD27^+^), and Effector memory (CD45RO^+^ CCR7^−^ CD27^−^) (**Figure 1**) (6). The desired memory CD4^+^ T cell population was sorted into tubes containing complete RPMI-1640 medium (10% FBS).

### 2.4 Post-sorting quality control

A small aliquot of sorted cells was re-analyzed on the FACS Aria III to confirm post-sort purity, which consistently exceeded 95%.

### 2.5 HIV-1 pro-viral DNA quantification

A 292 bp fragment encompassing a segment of the HIV-1 LTR and gag region was amplified from the DNA of an HIV-1 infected individual using nested PCR with primers sourced from previous literature (7). HIV-1 amplicon was cloned into an expression vector (PROMEGA, 2800 MADISON, WI USA) and the standards for the in-house quantitative assay were prepared.

DNA was extracted from sorted memory T cell subsets using the QIAcube system (Qiagen, Hilden, Germany) with the QIAcube HT Kit, according to the manufacturer’s instructions. The eluted DNA was used for quantification of HIV-1 pro-viral DNA. HIV-1 proviral DNA quantification was performed by amplifying a 158-bp fragment spanning portions of the long terminal repeat (LTR) and gag regions using primers F (ATCTCTAGCAGTGGCGCCCGA) and R (CCTTCTAGCCTCCGCTAGTCA). The amplified product was subsequently detected using the probe (ACGCAGGACTCGGCTTGCTG) (7). The cycling conditions were as follows, 95 °C for 10 minutes, followed by 95 °C for 30sec, 57 °C for 30 seconds, for 50 cycles.

An in-house ERV-3 (endogenous retrovirus) quantitative real time PCR was carried out to know the copy number in memory T cells (8). This assay is based on TaqMan chemistry, and the primers and probe were taken from previously published literature (9). The thermal cycling conditions used for this assay were 95 °C for 15 min, 95 °C for 45 sec, and 60 °C for 75 sec for 50 cycles The ERV-3 quantitation was done for the samples and the copy number calculated for the HIV-1pro-viral based on the ERV-3 copies per 10000 cells (10).

### 2.6 Statistical analysis

Baseline characteristics and clinical variables were summarized using frequencies and proportions for categorical data, and means with standard deviations or medians with interquartile ranges (IQR) for continuous data. Comparisons between immune responders and non-responders were made using the independent t-test or Mann-Whitney U test for continuous variables, and Pearson’s chi-square test or Fisher’s exact test for categorical variables. A two-tailed p-value of less than 0.05 was considered statistically significant. The proviral DNA copies among the subsets of memory CD4-positive T-cells were log-transformed to normalize distributions and facilitate visualization. We used STATA ^®^ version 16 (StataCorp. 2019. Stata Statistical Software: Release 16. College Station, TX: StataCorp LLC) for statistical analyses, and the figures were generated using GraphPad Prism.

## 3 Results

Among the 60 participants enrolled, 26 were classified as immunological non-responders and 34 as immunological responders. The majority were middle-aged (mean: 43.56 ± 10.22 years). The median duration of HIV infection was 10 years (IQR: 7–13), and the median duration on HAART was 7 years (IQR: 4–11). Zidovudine and tenofovir disoproxil fumarate (TDF) were the most frequently used drugs in baseline HAART regimens (43.34% and 41.67%, respectively). None of the participants had received integrase strand transfer inhibitors (INSTIs). Notably, an abacavir-based regimen was used exclusively in the INR group (30.77%) (**Table 2**).

**Table 2:** Baseline characteristics of immunological non-responders and immune responders.

Variables	Total (N = 60)	Immune non-responder (N = 26)	Immune responder (N = 34)	P-value
Age (mean ± SD)	43.56 ± 10.22	44.73 ± 11.07	42.67± 9.60	0.445
Gender (%) Male Female	29 (48.33) 31(51.67)	17 (65.38) 9(34.62)	12 (35.29) 22(64.71)	0.021
Comorbidities				
Diabetes Mellitus (%)	15 (25)	6 (23.08)	9 (26.47)	0.764
Systemic hypertension (%)	12 (20)	7 (26.92)	5 (14.71)	0.241
Coronary heart disease (%)	1 (1.67)	1 (3.85)	0	0.433
Chronic Smoking (%)	2 (3.33)	2 (7.69)	0	0.183
Chronic Alcoholism (%)	3 (5)	3 (11.54)	0	0.076
Others (%)	4 (6.67)	2^a^ (7.69)	2^b^ (5.88)	1.000
Mother to child transmission (%)	3 (5)	2 (7.69)	1 (2.94)	0.574
Baseline CD4 (median, IQR)	194.5 (111–328.5)	145.5 (105–261)	248 (140–443)	0.046
CD4 at the time of study (median, IQR)	529.5 (303.5–711.5)	299.5 (251–321)	688 (574–778)	<0.001
Duration Of HIV (in years) (median, IQR)	10 (7–13)	10.5 (7–14)	10 (7–13)	0.091
Duration Of ART (in years) (median, IQR)	7 (4–11)	8.5 (6–12)	7 (3–9)	0.092
Delay in ART (in years) (median, IQR)	1(0-5)	0(0-2)	1.5(0-5)	0.056
Combination anti-retroviral therapies				
Baseline Regimen *AZT- 3TC - EFV d4T -3TC- NVP AZT- 3TC- NVP TDF -3TC- EFV TDF – 3TC- NVP d4T – 3TC - EFV	10 (16.67) 7 (11.67) 16 (26.67) 24 (40) 1 (1.67) 2 (3.33)	4 (15.38) 3 (11.54) 11 (42.31) 7 (26.92) 0 1 (3.85)	6 (17.65) 4 (11.76) 5 (14.71) 17 (50) 1 (2.94) 1 (2.94)	0.162
Current Regimen AZT-3TC-EFV AZT-3TC-NVP TDF-3TC-EFV TDF -3TC-ATV/r ABC-3TC-ATV/r	1 (1.67) 4 (6.67) 36 (60) 11 (18.33) 8 (13.33)	0 2 (7.69) 13 (50) 3 (11.54) 8 (30.77)	1 (2.94) 2 (5.88) 23 (67.65) 8 (23.53) 0	0.003
Pre HAART opportunistic infections				
Pre ART OIS	23(38.33)	9 (34.62)	14 (41.18)	0.604
Tuberculosis (%)	16 (26.67)	8 (30.77)	8 (23.53)	0.530
Post HAART opportunistic infections				
Post ART OIS	12 (20)	7 (26.92)	5 (14.71)	0.241
Tuberculosis (%)	6 (10)	3 (11.54)	3 (8.82)	1.000
Death (%)	1 (1.67)	1^f^ (3.85)	0	0.433

HIV, Human immunodeficiency Virus; HAART, Highly active antiretroviral therapy; PCP, Pneumocystis pneumonia; a: 1. Small fiber neuropathy, 2. Dyslipidemia; b: 1. Dyslipidemia, 2. Vocal cord palsy of unknown etiology; c: Hodgkins Lymphoma; d: Diffuse Large B-cell Lymphoma; e: 1. Oral Candidiasis (INR), 2. Herpes labialis (INR), 3. Genital warts (IR); f: Cause of death is due to complications of extrapulmonary tuberculosis; *AZT, Zidovudine; 3TC, Lamivudine; EFV, Efavirenz; d4T, Stavudine; NVP, Nevirapine; TDF, Tenofovir disoproxil fumarate; ABC, Abacavir; ATV/r, Atazanavir/ritonavir.

Significant differences were observed between groups with respect to male gender, baseline CD4 count, and exposure to TDF-efavirenz (EFV)-based regimens. There were more male participants in the INR group than in the IR group (P = 0.021). The baseline CD4 counts and the CD4 counts during the study were significantly lower (P = 0.04 and P = <0.001) in the INR group (145.5 cells/µL with IQR of 105–261 and 299.5 cells/µL with IQR of 251–321, respectively) than in the IR group (248 cells/µL with IQR of 140–443 and 688 cells/µL with IQR of 574–778, respectively). Exposure to TDF-EFV-based regimens was higher in the IR group than in the INR group (23% versus 13%, P = 0.003). One participant from the INR group died due to complications related to tuberculosis.

**Figure 2:**
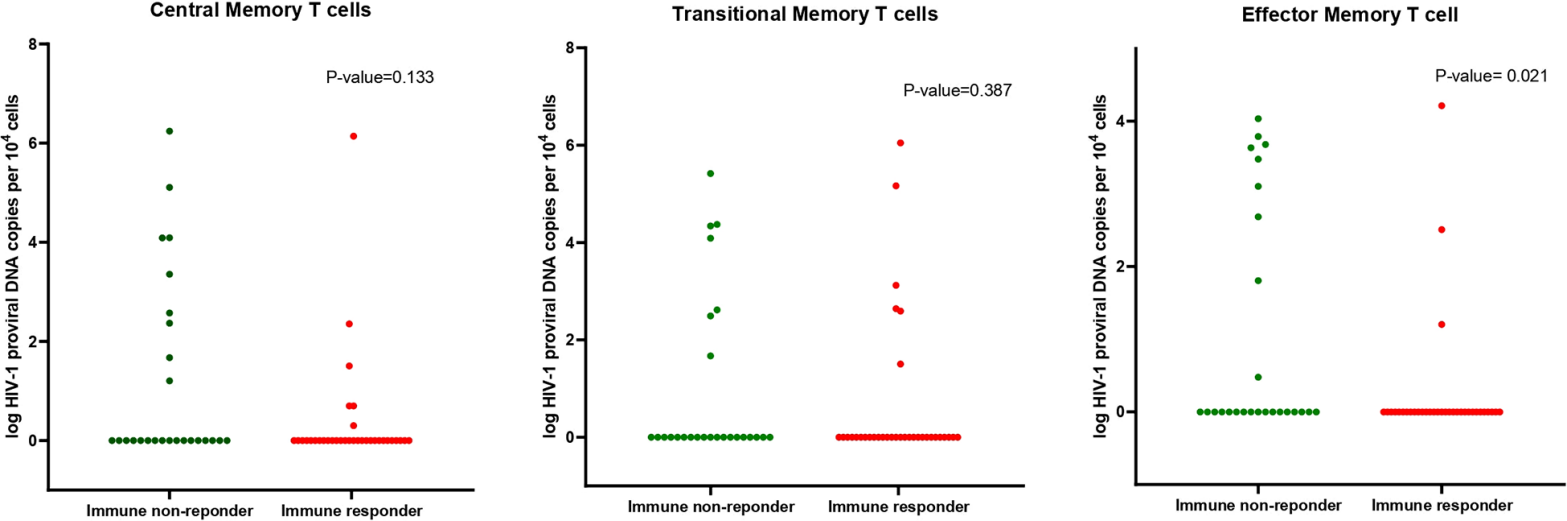
Distribution of HIV-1 proviral DNA copies in the INR and IR populations among memory T cell subsets.

Latent HIV reservoirs (LRs) were detected in at least one memory T cell subset in 48.33% of participants. Immunological non-responders demonstrated a significantly higher frequency of detectable LRs compared to IRs (65.38% versus 35.29%, P = 0.02) (**Table 3**). While latent reservoirs were observed across all memory subsets, the most common sites were central memory and effector memory T-cells. A statistically significant difference between groups was noted in the TEM subset, 34.6% of which had latent LRs in INRs, while 8.8% of the TEM subset had latent LRs in IRs (P = 0.02) (**Figure 2**). No statistically significant difference in LR was observed when stratified by baseline CD4 count, duration of HIV infection, or duration of HAART.

**Table 3:** Immune cell marker outcomes with 95% CI.

Variables	Immune non-responder (N = 26)	Immune responder (N = 34)	P-value
Latent reservoir (LR) in memory T cell subsets (%)	65.4% (47.09 – 83.67)	35.3% (19.23 – 51.35)	0.021
LR in Central memory T cells (TCM) (%)	34.6% (16.32– 52.90)	17.6% (4.83 – 30.46)	0.133
LR in Transitional memory T cells (TTM) (%)	26.9% (9.87 – 43.97)	17.6% (4.83 – 30.46)	0.387
LR in Effector memory T cells (TEM) (%)	34.6% (16.32 – 52.90)	8.8% (-0.71 – 18.35)	0.021

In TCM cells, the median (IQR) proviral DNA copies per microliter were 1,318 (47–12,342) in INRs versus 32 (5–225) in IRs (P = 0.198). For TTM cells, the medians were 12,262 (311–23,644) in INRs and 883 (392–145,738) in IRs (P = 1.000). In TEM cells, median values were 2,132 (64–4,774) in INRs and 323 (16–16,324) in IRs (P = 0.866). Although these differences did not reach statistical significance, the overall trend pointed toward higher proviral DNA levels in INRs across all memory subsets.

Additionally, participants receiving zidovudine- and nevirapine-based regimens exhibited a higher frequency of detectable LRs in memory T-cell subsets when compared to those on TDF-based regimens (**Table 2**).

## 4 Discussion

Our study demonstrated that INRs harbored a higher burden of HIV-1 proviral DNA within circulating memory CD4-positive T-cell subsets than IRs did. This provides novel evidence that the persistence of latent reservoirs in memory T cells is associated with poor CD4-positive T cell recovery despite long-term viral suppression with HAART. While other immune cells, such as regulatory T cells (Tregs), natural killer cells, Th17 cells, and polymorphonuclear myeloid-derived suppressor cells, have been implicated in immunologic failure in PLWH, memory CD4+ T cells could be the primary reservoir of latent HIV (11). Recent studies have demonstrated that peripheral blood monocytes in INRs carry a greater burden of HIV-1 RNA and DNA compared with IRs (12). Tissue-resident memory T cells (TRMs) have been shown to serve as significant HIV reservoirs and express higher levels of HIV susceptibility markers, such as CCR5 (13). Additionally, albeit smaller, contributions come from circulating mononuclear macrophages and B cells within lymphoid structures (14).

Among the memory CD4-positive subsets, central memory T cells are known to harbor the highest levels of HIV-1 proviral DNA, followed by transitional memory T cells, effector memory T cells, stem memory T cells, and naïve memory T cells (15). In our study, proviral DNA was detected across TCM, TTM, and TEM subsets, with a higher frequency of latent reservoirs in the TEM subset among INRs than among IRs. This observation may reflect the differing distributions and trafficking patterns of memory T cell subsets between peripheral blood and tissue compartments (16). Circulating CD4-positive T cells represent only 2–2.5% of the total T cell pool in the body, and over 90% of proviruses in these cells are replication-defective (17). Therefore, our findings may underestimate the true burden of replication-competent latent reservoirs. A more accurate assessment would require a quantitative viral outgrowth assay, which specifically measures inducible, replication-competent virus (18).

Immune non-responders, despite having virologic suppression, face a threefold increased risk of both AIDS-defining and non-AIDS-defining complications (19, 20). Although our study was underpowered to detect such statistical differences, herpes zoster, herpes labialis, and oral candidiasis occurred exclusively among INRs post HAART. Additionally, one INR developed diffuse large B-cell lymphoma, and another INR succumbed to extrapulmonary tuberculosis.

The impact of HAART on latent reservoirs remains an important area of investigation. Early initiation of antiretroviral therapy has been shown to reduce the reservoir size and may facilitate long-term viral control (21). Furthermore, certain antiretroviral classes, such as protease inhibitors and INSTIs, have demonstrated superior outcomes in terms of rapid viral suppression, better CD4 recovery, and lower rates of virological failure (22–26).

In our study, participants on zidovudine- and nevirapine-containing regimens had a higher frequency of latent reservoirs compared to those on TDF-based regimens. This may reflect the differing pharmacokinetic profiles and reservoir penetration capacities of various drug classes. For example, INSTIs may influence viral decay kinetics in resting memory T cells (27). This could not be evaluated in our study as none of our study participants had been exposed to INSTIs during or prior to the study period. Future pharmacokinetic modeling studies are warranted to better understand how newer HAART agents influence latent reservoir dynamics.

Overall, our findings highlight a significant burden of latent HIV in circulating memory CD4-positive T cells, with 48.33% of the study population harboring proviral DNA despite having prolonged viral suppression. This emphasizes the resilience of the latent reservoir and its continued role as a barrier to HIV eradication.

We acknowledge several limitations in this study. First, the observational design and small sample size limit generalizability and statistical power. Second, prolonged cryopreservation of PBMCs prior to sorting could have led to cell loss and the underestimation of reservoir size. Third, quantification of HIV-1 proviral DNA in circulating memory T cells provides only a partial view of the overall reservoir landscape. Assessing additional circulating subsets, such as circulating T follicular helper (cTfh) cells, may offer a comprehensive understanding of latent reservoirs.

## 5 Conclusion

Latent HIV reservoirs within memory CD4-positive T cells may significantly contribute to suboptimal immune recovery in immunological non-responders. Emerging classes of antiretroviral agents that target these latent viral pools hold promise for enhancing immune restoration and bringing us closer to the goal of a functional or sterilizing HIV cure.

## Data Availability

The raw data supporting the conclusions of this article will be made available by the authors, without undue reservation.
